# Higher Body Mass Index and Increased Prevalence of Paranasal Sinus Disease

**DOI:** 10.2188/jea.JE20150134

**Published:** 2016-05-05

**Authors:** Yusuke Kabeya, Kiyoe Kato, Masuomi Tomita, Takeshi Katsuki, Yoichi Oikawa, Akira Shimada

**Affiliations:** 1 Division of General Internal Medicine, Department of Internal Medicine, Tokai University Hachioji Hospital, Hachioji, Tokyo, Japan; 2 Department of Internal Medicine, Tokyo Saiseikai Central Hospital, Tokyo, Japan

**Keywords:** obesity, body mass index, paranasal sinus disease

## Abstract

**Background:**

We hypothesized that higher body mass index (BMI) was associated with increased prevalence of paranasal sinus disease and examined the hypothesis in Japanese adults.

**Methods:**

This was a cross-sectional study including 1350 Japanese adults aged 40 years or more who participated in a health check-up program focusing on brain diseases and metabolic syndrome. Participants were divided into quartiles of BMI levels. Paranasal sinus disease was confirmed by a head MRI scan. The association between BMI and paranasal sinus disease was examined using logistic regression analysis, which was adjusted for age, sex, waist:hip ratio, hemoglobin A1c, systolic blood pressure, smoking status, alcohol intake, and white blood cell count.

**Results:**

Of the 1350 participants, 151 (11.2%) had paranasal sinus disease. In relation to those in the lowest quartile of BMI, the odds ratios of having the disease among those in the 2nd, 3rd, and 4th quartiles of BMI were 1.89 (95% confidence interval [CI], 1.03–3.48), 2.26 (95% CI, 1.20–4.23) and 2.26 (95% CI, 1.14–4.51), respectively. When BMI was analysed as a continuous variable, an increase of one unit in BMI was significantly associated with increased odds of having the disease, with an OR of 1.08 (95% CI, 1.01–1.16).

**Conclusions:**

The present study suggests that patients with higher BMI are more likely to have paranasal sinus disease.

## INTRODUCTION

The epidemic of obesity prevails worldwide, along with changes in lifestyles and dietary patterns. The numbers of overweight and obese adults in 2005 were estimated to be 937 million and 396 million worldwide, which accounted for 23% and 10% of the world’s adult population, respectively.^1^ The estimated numbers of overweight and obese adults are projected to be 2.16 billion and 1.12 billion, respectively, by 2030. The increase would impose serious health burdens. The underlying pathophysiology of these conditions is characterized by chronic inflammation with excess adipose tissue,^2^^,^^3^ which could lead to the development of obesity-related diseases in various organ systems, including the respiratory system. Recently, several epidemiological studies have reported that higher BMI was associated with increased risk of asthma.^4^^–^^6^ Since asthma is considered to be a state of airway inflammation, it has been proposed that both conditions could have a common inflammatory pathway.^7^

Likewise, paranasal sinus disease, which is closely related to asthma according to the concept of unified airway,^8^^,^^9^ develops as a result of inflammation in the sinus cavities. Few studies have examined the association of obesity with paranasal sinus disease, especially among adults, with only one study^10^ examining large-scale data on the Medical Expenditure Panel Survey in the U.S. reporting an epidemiological link between obesity and paranasal sinus disease. The study concluded that obesity was significantly associated with increased risk of having chronic rhinosinusitis, with an odds ratio of 1.31.^10^ Although the study examined a large sample, the diagnosis of paranasal sinus disease was based on diagnosis codes. Difficulties in confirming a diagnosis of paranasal sinus disease might pose an obstacle to facilitating clinical research on the epidemiological link between paranasal sinus disease and obesity.

Previously, we performed a series of analyses^11^^,^^12^ investigating associations of glycemic status with lung functions and paranasal sinus disease in a sample of Japanese adults who underwent a health check-up program. The program included a head MRI scan, which enabled us to assess paranasal sinus disease in an objective way and provided the opportunity to examine the association between obesity and paranasal sinus disease. In the present study, we hypothesized that higher BMI was associated with increased prevalence of paranasal sinus disease and examined the hypothesis in a sample of Japanese adults.

## METHODS

### Study design and study population

This cross-sectional study was performed as part of analyses designed to investigate associations of brain diseases and paranasal sinus disease with other chronic diseases in a Japanese health check-up population. The study included 1351 Japanese adults aged 40 years or older who participated in a health check-up program focusing on metabolic syndrome and brain diseases from January 2007 to December 2011. Participants were apparently healthy and visited the hospital not for symptomatic diseases but for the purpose of a health check-up. One participant was excluded because of missing data. As a result, 1350 participants were included in the analysis. The study protocol was reviewed and approved by the ethics committee of Saiseikai Central Hospital.

### Anthropometric and laboratory measurements

Since the present study was performed as part of a larger set of analyses, the detailed methods of collecting anthropometric and laboratory data have been reported elsewhere.^11^^,^^12^ In brief, information on age, sex, weight, height, body mass index (BMI), waist:hip ratio, blood pressure, white blood cell (WBC) count, fasting plasma glucose levels, and hemoglobin A1c (HbA1c) levels was collected. Information on smoking status and alcohol intake was obtained using a self-administered questionnaire. As for smoking status, participants were sorted into three groups (non-smoker, past smoker, or current smoker). With regard to alcohol intake, they were categorized into three groups according to frequency of alcohol intake (abstainer, 1–4 days per week, or 5 days or more per week).

### Paranasal sinus disease

In the present study, paranasal sinus disease was diagnosed if a participant had any abnormal findings (mucosal thickening, polyps, or sinus opacification) in at least one of the paranasal sinuses. The criteria were established by referencing the Lund-Mackay system,^13^ which is a widely accepted method for grading paranasal sinus abnormalities.^14^ In the Lund-Mackay system,^13^ paranasal sinus abnormality was categorized as normal, partially opacified (including fluid retention, polyps, and mucosal thickening), or totally opacified. The findings were detected via head MRI scan with a 1.5-Tesla scanner (SIGNA HDxt 1.5T; GE Healthcare Japan, Tokyo, Japan). The images were assessed by trained neurologists who did not know the purpose of the study.

### Statistical analysis

The participants were divided into quartiles of BMI levels according to the study population distribution. Characteristics of the participants were compared across the BMI categories. One-way analysis of variance tests were used for comparing continuous variables, while χ^2^ tests were used for categorical variables. Regarding WBC count, the log-transformed values were compared because of the skewed distribution of WBC count. Then, the association between BMI and the prevalence of paranasal sinus disease was graphically and statistically examined. The observed prevalence of paranasal sinus disease for each 0.4 unit of BMI band was plotted, and the expected prevalences of paranasal sinus disease and their 95% confidence intervals (CIs) in each BMI level were plotted in the same graph using a restricted cubic spline model. The model confirmed that the log-odds of having paranasal sinus disease were a linear function of BMI level. Finally, the odds ratios (ORs) and 95% CIs of having paranasal sinus disease were estimated for quartiles of BMI levels using a multiple logistic regression model. The ORs and the 95% CIs were adjusted for age, sex, waist:hip ratio, HbA1c, systolic blood pressure, smoking status, alcohol intake, and WBC count. To test for linear trend, the BMI data were scored from 1 (lowest) to 4 (highest) quartile. Then, trend tests were performed by modelling these quartile-based scores as a continuous variable. In the multiple logistic regression, we did not stratify the analysis by sex, since no significant effect modification of the association by sex was observed. In this process, models with and without the interaction term of sex and BMI were constructed and compared using a chi-squared log-likelihood ratio test (χ^2^ = 0.47, *P* = 0.49), which found no significant effect modification.

Statistical analyses were performed using STATA software version 11 (StataCorp, College Station, TX, USA). All statistical tests were two-sided, and *p*-values less than 0.05 were considered statistically significant.

## RESULTS

Among the 1350 participants, paranasal sinus disease was detected in 151 participants (11.2% of the overall population). Table 1 shows the prevalence of paranasal sinus disease and other characteristics of the study participants by quartile of BMI. The prevalence of paranasal sinus disease differed significantly across the quartiles. Prevalence increased with the BMI quartiles, and sex ratio, waist:hip ratio, systolic blood pressure, diastolic blood pressure, fasting plasma glucose, HbA1c, and WBC count were also significantly different across the quartiles. The differences in age and alcohol intake were significant but less pronounced.

**Table 1.** Characteristics of study participants

	Total	Quartile of BMI				*P* for difference

		1	2	3	4	
BMI range, kg/m^2^		15.2–21.3	21.3–23.1	23.1–25.3	25.3–36.5	
BMI mean, kg/m^2^	23.4 (3.2)	19.6 (1.3)	22.2 (0.5)	24.2 (0.6)	27.5 (2.2)	
Number of participants	1350	338	337	338	337	
Paranasal sinus disease, *n* (%)	151 (11.2)	21 (6.2)	38 (11.3)	46 (13.6)	46 (13.7)	<0.001

Age, years	61.6 (10.0)	61.1 (10.6)	62.6 (9.8)	62.2 (10.1)	60.5 (9.5)	0.023
Males, *n* (%)	966 (71.6)	139 (41.1)	257 (76.3)	288 (85.2)	282 (83.7)	<0.001
Waist:hip ratio	0.90 (0.07)	0.84 (0.07)	0.90 (0.05)	0.92 (0.05)	0.95 (0.05)	<0.001
Systolic blood pressure, mm Hg	122 (19)	117 (18)	122 (19)	125 (18)	127 (18)	<0.001
Diastolic blood pressure, mm Hg	77 (12)	73 (11)	75 (11)	77 (10)	81 (12)	<0.001
Fasting plasma glucose, mg/dL	107 (23)	100 (21)	105 (21)	108 (22)	113 (25)	<0.001
HbA1c, %	5.8 (0.8)	5.6 (0.6)	5.8 (0.7)	5.9 (0.8)	6.0 (0.8)	<0.001
WBC^a^, count/µL	5400 (5320–5470)	5030 (4890–5170)	5180 (5040–5330)	5450 (5300–5600)	5970 (5810–6140)	<0.001
Smoking status, *n* (%)						
non-smoker	764 (56.6)	231 (68)	185 (55)	178 (53)	170 (50)	<0.001
past smoker	417 (30.9)	71 (21.0)	107 (32)	123 (36)	116 (34)	
current smoker	169 (12.5)	36 (11)	45 (13)	37 (11.0)	51 (15)	
Alcohol intake, *n* (%)						
abstainer	273 (20.2)	87 (26)	64 (19.0)	62 (18)	60 (18)	0.024
1–4 days per week	620 (45.9)	144 (43)	160 (47)	144 (43)	172 (51.0)	
5 days or more per week	457 (33.9)	107 (32)	113 (34)	132 (39)	105 (31)	

BMI, body mass index; HbA1c, hemoglobin A1c; WBC, white blood cell.

Data are presented as mean (standard deviation) for continuous variables and count (%) for categorical variables except WBC.

^a^WBC count is presented as geometric mean (95% confidence interval) because of the skewed distribution.

The prevalence of paranasal sinus disease was further investigated using a restricted cubic spline model, which showed a positive association between BMI levels and the presence of paranasal sinus disease, although the association appeared attenuated in those with high BMI levels (Figure).

**Figure.**
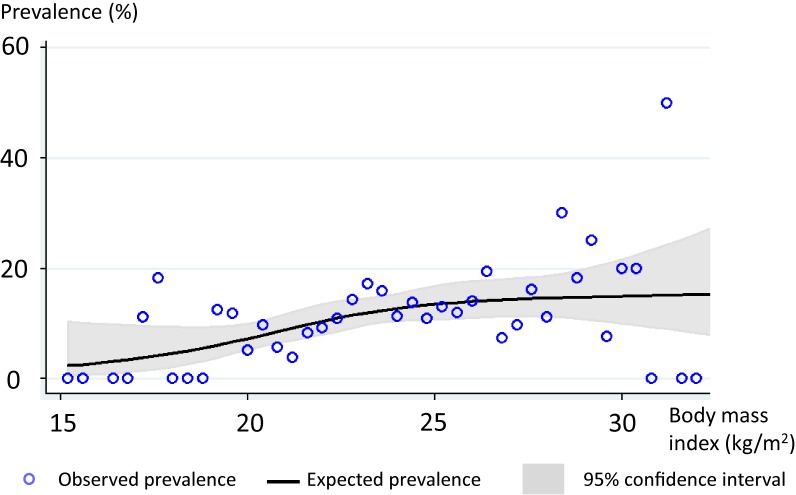
The prevalence of paranasal sinus disease according to body mass index.

Table 2 shows the result of the logistic regression analysis. Although the variables listed in Table 2 produced significance or borderline significance in the crude analysis, the significant associations remained in the multivariate analysis only for age, BMI, and HbA1c levels. In relation to those in the lowest quartile of BMI, the odds of having paranasal sinus disease were significantly higher among those in the 2nd, 3rd, and 4th quartiles of BMI, with ORs of 1.89 (95% confidence interval [CI], 1.03–3.48), 2.26 (95% CI, 1.20–4.23) and 2.26 (95% CI, 1.14–4.51), respectively. A significant linear trend was observed (*P* for trend = 0.034). When BMI was analysed as a continuous variable, an increase of one unit of BMI was significantly associated with increased odds of having paranasal sinus disease, with an OR of 1.08 (95% CI, 1.01–1.16).

**Table 2.** The odds ratios of having paranasal sinus disease according to body mass index

	Crude OR	95% CI	Adjusted OR^a^	95% CI	Adjusted OR^b^	95% CI
BMI quartile						
1st	1.00	reference	1.00	reference		
2nd	1.92	1.10–3.34	1.89	1.03–3.48		
3rd	2.38	1.39–4.08	2.26	1.20–4.23		
4th	2.39	1.39–4.10	2.26	1.14–4.51		
	(*P* for trend = 0.001)		(*P* for trend = 0.034)			

BMI (increase of 1 unit)	1.09	1.03–1.14			1.08	1.01–1.16

Age (increase of 10 years)	0.82	0.69–0.98	0.80	0.66–0.97	0.82	0.67–1.00

Sex						
men	1.00	reference	1.00	reference	1.00	reference
women	0.51	0.33–0.79	0.74	0.44–1.24	0.66	0.40–1.11

Systolic blood pressure (increase of 10 mm Hg)	1.03	0.94–1.12	1.02	0.92–1.12	1.01	0.92–1.12

HbA1c (increase of 1%)	1.25	1.03–1.52	1.30	1.05–1.61	1.28	1.03–1.59

Waist:hip ratio (increase of 0.10)	1.29	0.99–1.67	0.81	0.55–1.19	0.80	0.54–1.19

log WBC count (increase of 1 standard deviation)	1.16	0.98–1.37	1.03	0.85–1.24	1.01	0.84–1.22

Smoking status						
non-smoker	1.00	reference	1.00	reference	1.00	reference
past smoker	1.24	0.85–1.82	1.09	0.74–1.61	1.10	0.74–1.64
current smoker	1.85	1.16–2.97	1.42	0.85–2.38	1.43	0.85–2.40

Alcohol intake						
abstainer	1.00	reference	1.00	reference	1.00	reference
1–4 days per week	1.09	0.66–1.79	0.92	0.55–1.54	0.91	0.54–1.54
5 days or more per week	1.81	1.11–2.97	1.58	0.93–2.68	1.60	0.94–2.72

BMI, body mass index; CI, confidence interval; HbA1c, hemoglobin A1c; OR, odds ratio; WBC, white blood cell.

^a^The model included BMI (categorical variable), age, sex, systolic blood pressure, HbA1c, waist:hip ratio, WBC count, smoking status, and alcohol intake.

^b^The model included BMI (continuous variable), age, sex, systolic blood pressure, HbA1c, waist:hip ratio, WBC count, smoking status, and alcohol intake.

## DISCUSSION

The present study examined the association between BMI levels and the prevalence of paranasal sinus disease in a Japanese health check-up population. Overall, we found a positive association between BMI levels and the prevalence of paranasal sinus disease. In addition, we found that younger age and higher HbA1c levels were significantly associated with increased prevalence of paranasal sinus disease.

The multivariate analysis demonstrated that the association of BMI with paranasal sinus disease remained significant after adjustment for HbA1c levels. Since BMI and glycemic status are closely related to each other, the apparent association of higher BMI with paranasal sinus disease may be confounded by glycemic status. However, the results showed that both HbA1c and BMI were significantly and independently associated with paranasal sinus disease. Although there may be some overlap in the mechanisms of the association, the results imply that higher glycemic status and higher BMI might affect paranasal sinus disease in different manners.

The multivariate analysis also showed that the association of BMI with paranasal sinus disease was independent of waist:hip ratio, which is a marker of central obesity.^15^ According to a past report,^16^ body fat distribution, which reflects systemic inflammation, may be a more accurate measure of obesity than BMI. We expected that the association of BMI with paranasal sinus disease became attenuated after adjustment for the waist:hip ratio in the present study. However, the association did not change substantially after such adjustment. This finding suggests that the link between higher BMI and paranasal sinus disease was not necessarily mediated through inflammation. Other mechanisms, such as airway structural problems related to obesity or obesity-related lifestyles, could mediate the association. Of particular interest is the shape of the curve in the plot of the dose-response relationship between BMI and the prevalence of paranasal sinus disease. The positive association appeared attenuated in those with high BMI levels. Although the reason for the attenuation remains uncertain, this finding could provide an insight into the mechanism of the association.

Since confirming the diagnosis of paranasal sinus disease seems difficult without objective imaging methods, the etiology, pathophysiology, and natural history of the disease have not been fully elucidated. Nonetheless, the clinical characteristics related to paranasal sinus disease have been investigated in several studies.^10^^,^^17^^–^^20^ However, results have been inconsistent. As chronic sinusitis is frequently diagnosed in young or middle-aged adults,^20^ it is expected that the prevalence of paranasal sinus disease decreases with age, which seems consistent with the findings of the present study. However, some studies^17^^–^^19^ have reported that age is not associated with the findings of head MRI scans assessing the paranasal sinuses. Evidence on the influence of HbA1c levels on paranasal sinus disease is limited. We previously reported that diabetes is associated with increased prevalence of paranasal sinus disease.^12^ The dose-response relationship between glycemic status and the prevalence was also confirmed in the study. Factors related to higher blood glucose levels, such as impaired immune function or impaired ciliary motility, could affect the development of paranasal sinus disease. Smoking is considered an established risk factor for chronic sinusitis.^21^ However, past studies^17^^–^^19^ using MRI reported that smoking habit did not correlate with abnormal findings in the paranasal sinuses, which is consistent with the results of the present study. These controversies should be resolved in future studies.

Although fatal consequences are not usually expected from paranasal sinus disease, the disease can result in serious healthcare costs. In the United States, chronic rhinosinusitis was considered to be one of the top 10 most costly conditions for American employers.^22^ The symptoms and impairment of quality of life resulting from rhinosinusitis resulted in 11.1 million healthcare visits per year, and the healthcare costs were estimated to be $8.6 billion in 2007.^23^^,^^24^ When indirect costs of the disease are taken into account, the overall costs related to the disease may be even higher. The healthcare costs of patients with chronic rhinosinusitis were also examined in an Asian population. One study in Taiwan^25^ reported that those with chronic rhinosinusitis visited outpatient clinics more frequently. Higher total healthcare costs among those with chronic rhinosinusitis compared with those without were also reported. There have been few studies reporting healthcare costs of paranasal sinus disease in Japan. As the prevalence of obesity has risen dramatically,^1^ it is expected that the prevalence of paranasal sinus disease is increasing, which might result in heavy healthcare cost burden. Further research is required to determine accurate epidemiology and healthcare cost estimates related to paranasal sinus disease.

A few limitations of the present study should be noted. First, the study was based on incidental findings of a head MRI scan. The clinical relevance of incidental findings detected by a head MRI scan has been controversial. One study^17^ demonstrated a correlation between abnormalities on a head MRI scan and symptoms of an upper respiratory infection. Other studies^26^^,^^27^ have reported that there is a disparity between clinical symptoms and the findings of radiological examination. Examining the association of BMI with clinically-confirmed paranasal sinus disease might be a worthwhile subject for a further study. Second, the presence of paranasal sinus disease was evaluated by trained neurologists. Although they were experienced in interpreting head MRI images, the between-observers variability was not available in the analysis, which could limit the validity of the present study. Third, selection bias is another concern, since the present study was performed among those who participated in a health check-up program. In general, those who voluntarily participate in a health check-up program might be more health-conscious than the general population, which could restrict the external validity of the present study. Fourth, although an association between higher BMI and increased prevalence of paranasal sinus disease was observed in the present study, the association between BMI and disease severity was not evaluated. In addition, we are not able to evaluate the causal association of BMI and paranasal sinus disease because of the cross-sectional design of the present study.

In conclusion, the present study found that higher BMI was associated with increased prevalence of paranasal sinus disease. This result has implications at both the individual and societal levels. At the individual level, physicians should remember that an obese patient may be at increased risk for paranasal sinus disease. At the societal level, it is expected that the prevalence of paranasal sinus disease is increasing with the epidemic of obesity, which could impose a substantial healthcare costs burden. Further research is required to better understand the epidemiology of paranasal sinus disease and the link between obesity and paranasal sinus disease.
